# Lateral meniscus posterior root repair during ACL reconstruction restores knee stability and results in outcomes comparable to isolated ACL reconstruction: A propensity‐matched cohort analysis

**DOI:** 10.1002/jeo2.70725

**Published:** 2026-04-14

**Authors:** Nikolaos Koukoulias, George Mihai Avram, Evangelia Ioannis Germanou, Dimitris Koukoulias, Georgios Graikos, Angelo Vasiliadis, Ioannis Tsifountoudis, Theofilos Dimitriadis

**Affiliations:** ^1^ Sports Trauma and Orthopaedics Department Saint Luke′s Hospital Thessaloniki Greece; ^2^ Department of Clinical Research, Research Group Michael T. Hirschmann, Regenerative Medicine & Biomechanics University of Basel Basel Switzerland; ^3^ Central Military Emergency Hospital Dr. Carol Davila Bucharest Romania; ^4^ Department of Physical Education and Sports Science Aristotle University of Thessaloniki Thessaloniki Greece; ^5^ Aristotle University of Thessaloniki Thessaloniki Greece; ^6^ Department of Radiology 424 General Military Training Hospital Thessaloniki Greece

**Keywords:** ACL reconstruction, knee stability, lateral meniscus posterior root tear, meniscal extrusion, meniscal healing

## Abstract

**Purpose:**

To compare clinical and MRI outcomes as well as knee anterior‐posterior (AP) and rotational stability between ACL reconstructions (ACLR) with concomitant lateral meniscus posterior root (LMPR) repair and matched isolated ACLR, while assessing whether different LMPR tear patterns affect post‐operative stability, healing or meniscal extrusion.

**Methods:**

A retrospective review was performed on patients < 55 years old who underwent ACLR and LMPR repair between 2018 and 2022, with minimum 2‐year follow‐up. Patients with osteoarthritis, prior meniscal or cartilage surgery, revision ACLR, or multi‐ligament injuries were excluded. The LMPR group was stratified into oblique radial (I), longitudinal/T‐type (II), and avulsion (III) tears. A 2:1 propensity score‐matched control cohort of isolated ACLR was created. Pre‐ and post‐operative knee stability (pivot shift, KT‐1000), PROMs (IKDC, Lysholm, Tegner), LMPR healing, lateral meniscus extrusion (LME), and MRI parameters (posterior meniscofemoral ligament) pMFL, (lateral femoral notch sign) LFNS were analysed.

**Results:**

Forty‐one LMPR repairs and 20 matched controls were included. Pre‐operative instability was similar between groups (pivot shift *p* = 0.09, KT‐1000 *p* = 0.92). Significant improvements in pivot shift and KT‐1000 were observed post‐operatively in both groups (all *p* < 0.001), with no post‐operative differences between them (pivot shift *p* = 0.72, KT‐1000 *p* = 0.94). PROMs improved significantly in all groups (*p* < 0.001) with no post‐operative group differences. Post‐operative reduction of LME did not differ statistically among tear types (*p* > 0.05), but LME still remained slightly higher in the study group. LMPR healing was high (75.6% complete, 24.4% partial) with no failures and no difference in healing across tear patterns (*p* = 0.46). The presence of LFNS or pMFL was not associated with tear type or with LMPR versus control status (*p* > 0.05).

**Conclusion:**

LMPR repair performed during ACLR successfully restores knee stability, decreases LME, and achieves functional outcomes similar to isolated ACLR. Tear morphology does not significantly affect post‐operative clinical or magnetic resonance imaging results.

**Level of Evidence:**

Level III, retrospective comparative study.

**Abbreviations**
ACL: anterior cruciate ligament
ALRI: antero‐lateral rotatory instability
AP: anterior‐posterior
GCP: good clinical practice
IKDC: International Knee Documentation Committee
LEAP: lateral extra‐articular procedures
LET: lateral extra‐articular tenodesis
LFC: lateral femoral condyle
LFNS: lateral femoral notch sign
LME: lateral meniscus extrusion
LMPR: lateral meniscus posterior root
LMPRT: lateral meniscus posterior root tear(s)
LTC: lateral tibial condyle
MRI: magnetic resonance imaging
OA: osteoarthritis
pMFL: posterior meniscofemoral ligament
PROMs: patient‐reported outcome measures
ROM: range of motion

## INTRODUCTION

Lateral meniscus posterior root (LMPR) tears (LMPRT) are increasingly recognised in the setting of anterior cruciate ligament (ACL) injuries. As a direct consequence, in an ACL‐deficient knee, a tear of the LMPR worsens rotational stability under pivot‐shift loading [35] and increases lateral tibial translation [11, 35]. Due to the native laxity of the knee′s lateral compartment and it′s characteristic biomechanics during knee flexion, internal tibial rotation combined with external rotation of the femoral condyle, which characterise both the screw‐home mechanism as well as posterior femoral roll‐back, a LMPRT will also alter the load distribution across the lateral compartment leading to an up to 40% contact pressure increase [29, 32], which has been shown to have long‐term consequences [14].

Although several patterns of LMPRT have been described, no universally accepted classification exists. The most common categorisation is based on arthroscopic morphology [2, 13, 22, 23]. Despite these efforts, the clinical and radiographic implications, specifically concerning lateral meniscus extrusion (LME), remain unclear and whether different LMPRT morphologies carry distinct biomechanical or prognostic consequences in still debated.

Clinically, an ACL‐deficient knee with a concomitant LMPRT typically demonstrates anterolateral rotatory instability (ALRI) [28], characterised by a higher‐grade pivot shift, increased lateral tibial translation, LME [31, 47], and a higher likelihood of a prominent lateral femoral notch sign (LFNS) on magnetic resonance imaging (MRI) [5, 15]. Additionally, the integrity of the posterior meniscofemoral ligament (pMFL) should be carefully evaluated on MRI, as it shares a reciprocal load‐bearing and force‐transmission relationship with the LMPR throughout the knee′s range of motion [30, 33].

Given the documented biomechanical alterations associated with ALRI and long‐term implications of MRI abnormalities within the context of concomitant ACL rupture and LMPRT, further characterisation of their clinical impact during ACLR is essential. Therefore, the primary aim of this study was to evaluate clinical outcomes and knee stability in patients undergoing combined ACLR with concomitant LMPRT repair, and to compare these results with those of patients undergoing isolated ACLR. A secondary aim was to determine whether different LMPRT subgroups influenced post‐operative knee stability testing or PROMs. The tertiary aim was to compare the extent of LME between groups, assess extrusion reduction at follow‐up, evaluate LMPRT repair healing rates, and examine the presence of a LFNS and pMFL in both cohorts. The primary hypothesis was that concomitant ACLR and LMPRT repair would lead to clinical outcomes and knee stability at follow‐up comparable to those of the reference group, isolated ACLR.

## METHODS

The employed methodology was that of a retrospective review of prospectively collected data of patients with ACLR and concomitant LMPRT that required repair. Data collection has been performed between January 2018 and December 2022. STROBE guidelines were followed and the study was conducted in accordance with the Declaration of Helsinki, GCP standards, and approved by the institutional review board; informed consent was obtained by all participants in this study.

### Inclusion criteria were

1. Patients <55 years old that underwent concomitant ACLR and LMPRT repair.
2. Absence of advanced osteoarthritic changes (Kellgren–Lawrence Grade 3 or 4).
3. Pre‐operative and post‐operative MRI images.
4. Pre‐ and post‐operative patient reported outcome measures (PROMs) (International Knee Documentation Committee [IKDC], Lysholm, and Tegner Activity Scale).
5. More than 2 years of follow‐up.

### Exclusion criteria were

1. Patients with stable LMPRT that did not require repair.
2. Revision ACLR.
3. Multi‐ligament knee injuries that necessitate repair or reconstruction of other. ligaments in addition to ACLR.
4. Prior meniscal surgery.
5. Prior or concomitant cartilage surgery.

After applying inclusion and exclusion criteria, 41 patients were eligible for inclusion. These patients represented the study group and were matched using a 2:1 matching protocol to 20 patients with isolated ACLR, representing the control group (details of the matching process are described in the *Statistical analysis* section). The study group was further categorised, according to the tear pattern, to *subgroup I*, oblique tear, *subgroup II*, longitudinal or “T” shape tear, and *subgroup III*, soft tissue or bony avulsion injury [45]. Due to unsatisfactory results when the LMPRT is left unaddressed [34], the authors of the present study had a low threshold for repairing LMPRT. During arthroscopic examination, LMPRT were probed to assess stability. Oblique, longitudinal, or “T” tears were less often completely unstable, although some degree of instability was present in all cases; these were generally repaired using an all‐inside technique. In contrast, Type III tears were typically unstable and were therefore treated with a pull‐out repair technique. Knee stability (pivot shift and KT‐1000), patient reported outcome measures, healing rate, reoperation rate, LME and presence of LFNS and pMFL were studied and compared between the two groups.

### Data collection

**Figure 1:**
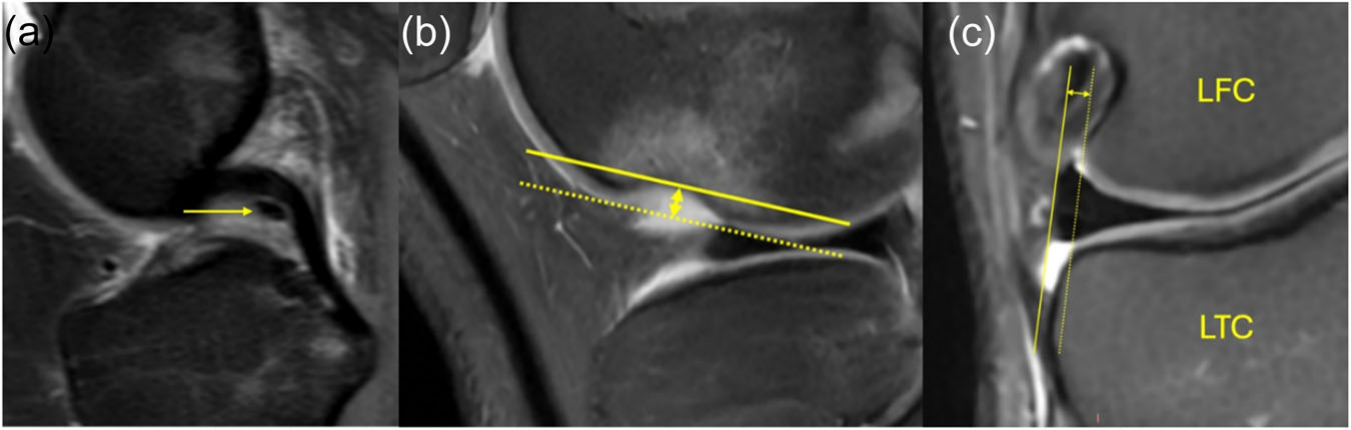
Investigated MRI signs. (a) pMFL presence; (b) deep LFNS; (c) LME. LFC, lateral femoral condyle; LFNS, lateral femoral notch sign; LME, lateral meniscus extrusion; LTC, lateral tibial condyle; MRI, magnetic resonance imaging; pMFL, posterior meniscofemoral ligament.

Surgical images and arthroscopic recordings were used to evaluate the LMPRT pattern. Patients were categorized accordingly into Group I, Group II or Group III. Pre‐operative and post‐operative data of all included patients were collected from medical records, patient reported clinical measures and pivot shift, and pre‐ and post‐operative MRIs. Specifically, the Subjective IKDC score [3] and Lysholm score [27] were recorded before surgery and at last follow‐up (defined as ≥2 years), while Tegner Activity Scale [39] was recorded *before injury*, *pre‐operatively*, and *post‐operatively* (defined as ≥2 years). All PROMs were collected during routine clinical visits by a study nurse, during pre‐clinical assessment. Pre‐operative MRI films were evaluated for the presence of the pMFL, deep LFNS of ≥2 mm [15], and extent of maximal lateral meniscus extrusion in the mid coronal plane [9] (Figure 1). These measurements have been previously shown to have reliable detection and reproducible determination [8, 17, 25]. Imaging measurements were performed by a single musculoskeletal radiologist with more than 15 years of experience, who was blinded to the study aims. The depth of the lateral femoral notch was evaluated in sagittal images and defined as the distance in mm from the deepest part of the sulcus of the lateral femoral notch to a tangent line that connects the anterior and posterior edges of the notch. For the sign to be deemed present, a minimum depth of 2.0 mm was considered necessary [15]. For LME, the slice with maximum extrusion in the midcoronal plane was used for the measurement. A line joining the most lateral point of lateral tibial condyle cartilage and lateral femoral condyle cartilage was drawn. Then, a second line was drawn at the level of the menisco‐capsular junction, parallel to the first one. The distance between those two lines was considered the LME [9]. The presence of MFLs was noted as thin, linear structures that exhibit low MR signal intensity on coronal slices. In the sagittal plane MFLs appear as dot‐like structures anterior or posterior to the posterior cruciate ligament [6]. All patients underwent a 1.5 T MRI scan. The MRI machine could not be controlled given that patients performed their scans in different locations. Images were first screened for motion artifacts—this was the sole exclusion criterion that warranted the images unusable.

Post‐operative MRI films were used to assess meniscal healing and possible reduction of lateral meniscus extrusion at 9–12 months after surgery following the methodology outlined by Cuvillier et al. [9]. State of meniscal healing was classified as: (a) complete healing, identifiable meniscus and normal meniscus signal in both coronal and sagittal planes, (b) partial healing, characterised by the absence of an identifiable meniscus or abnormal signal in either the coronal or sagittal plane and (c) failure of healing, no identifiable meniscus in both coronal and sagittal planes. This method has been used for both side‐to‐side and transtibial pull‐out repairs of LMPRT [19, 46, 47].

**Figure 2:**
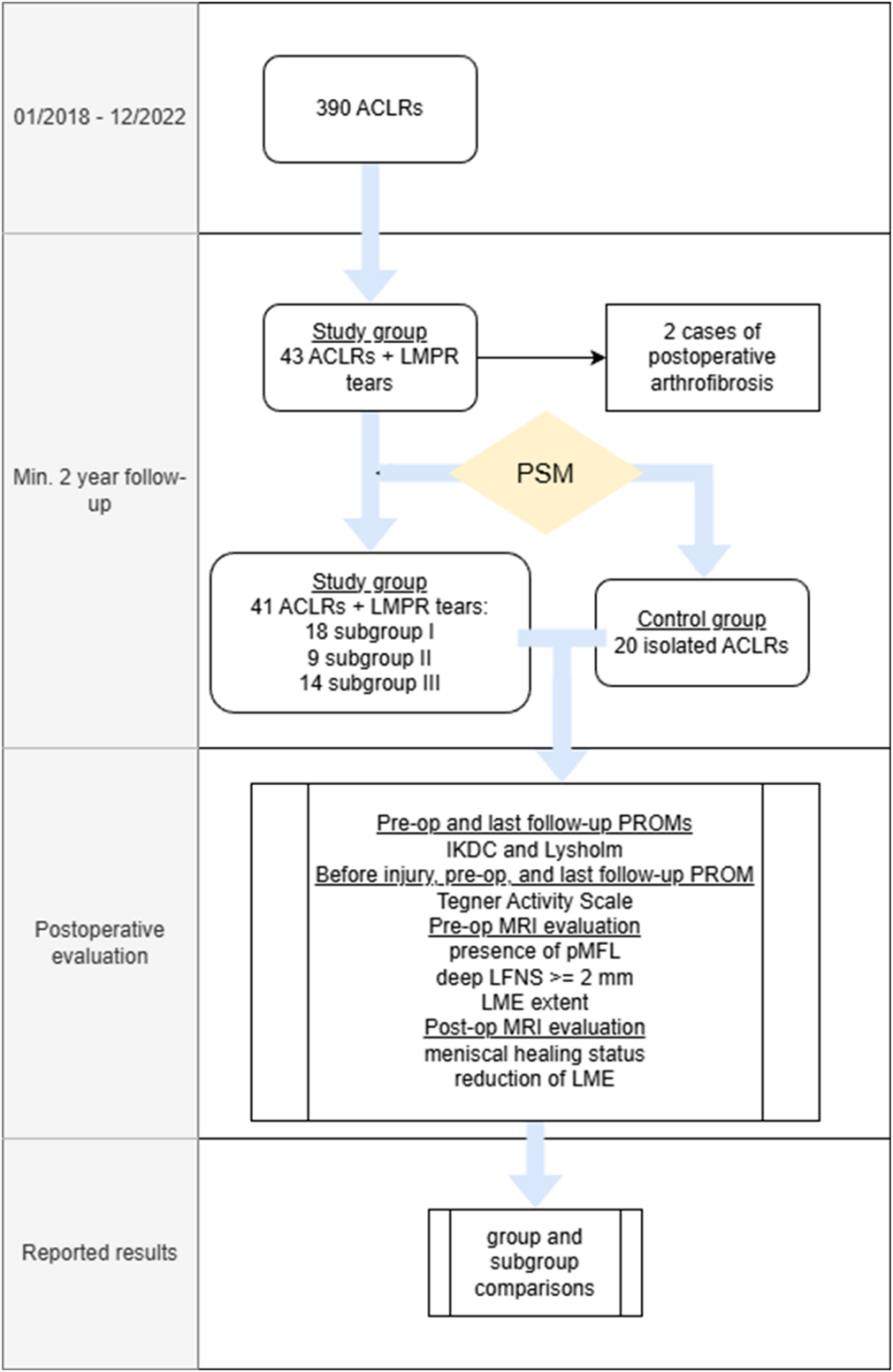
Flow diagram of the employed methodology. ACLRs, anterior cruciate ligament reconstructions; IKDC, International Knee Documentation Committee; LFNS, lateral femoral notch sign; LME, lateral meniscus extrusion; LMPR, lateral meniscus posterior root; MRI, magnetic resonance imaging; pMFL, posterior meniscofemoral ligament; PROM, patient‐reported outcome measure.

Figure 2 outlines the employed methodological structure.

### Surgical technique

All surgeries were performed by the same, fellowship‐trained, surgeon with extensive experience in treating knee sports injuries. A single bundle, anatomic ACLR was performed in all cases with hamstrings, quadriceps or patellar tendon graft. A lateral extra‐articular procedure (LEAP) (in this case, LET) was performed in high‐risk patients (age <25 years old, professional athletes, pivoting sports and hyperextension ≥5°). Medial meniscus pathology was addressed with partial meniscectomy in irreparable tears and meniscus repair in all other tears.

For ACLR, the femoral and tibial tunnels were prepared first. In case of a LMPRT pull‐out technique, the tibial tunnel for the root was drilled right after this step. Subsequently the ACL graft was passed but not fixed. The LMPRT was then repaired (and fixed with a button only when a pull‐out technique was required), followed by ACL graft fixation.

**Figure 3:**
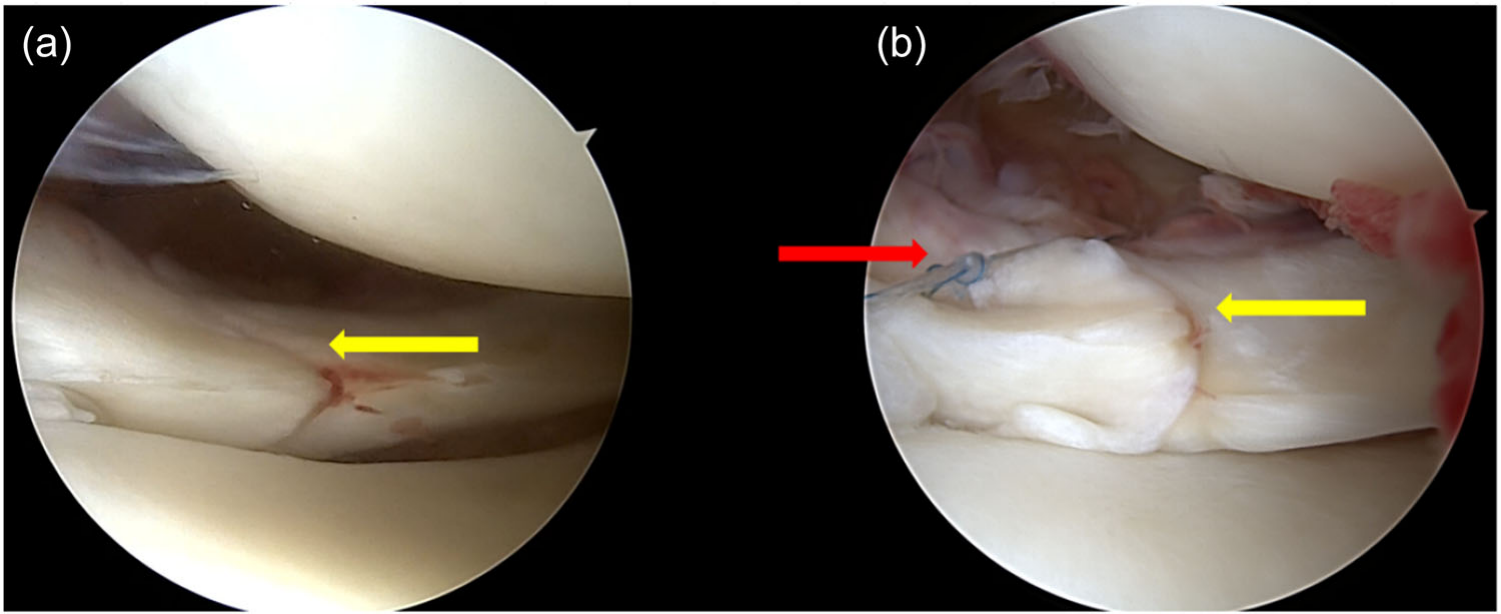
Arthroscopic evaluation of oblique tears. (a) Arthroscopic assessment of tear pattern (yellow arrow). (b) Oblique tear (yellow arrow) after all‐inside suture repair (red arrow).

Oblique tears (Group I) were repaired with all‐inside sutures in a side‐to‐side fashion (Figure 3).

**Figure 4:**
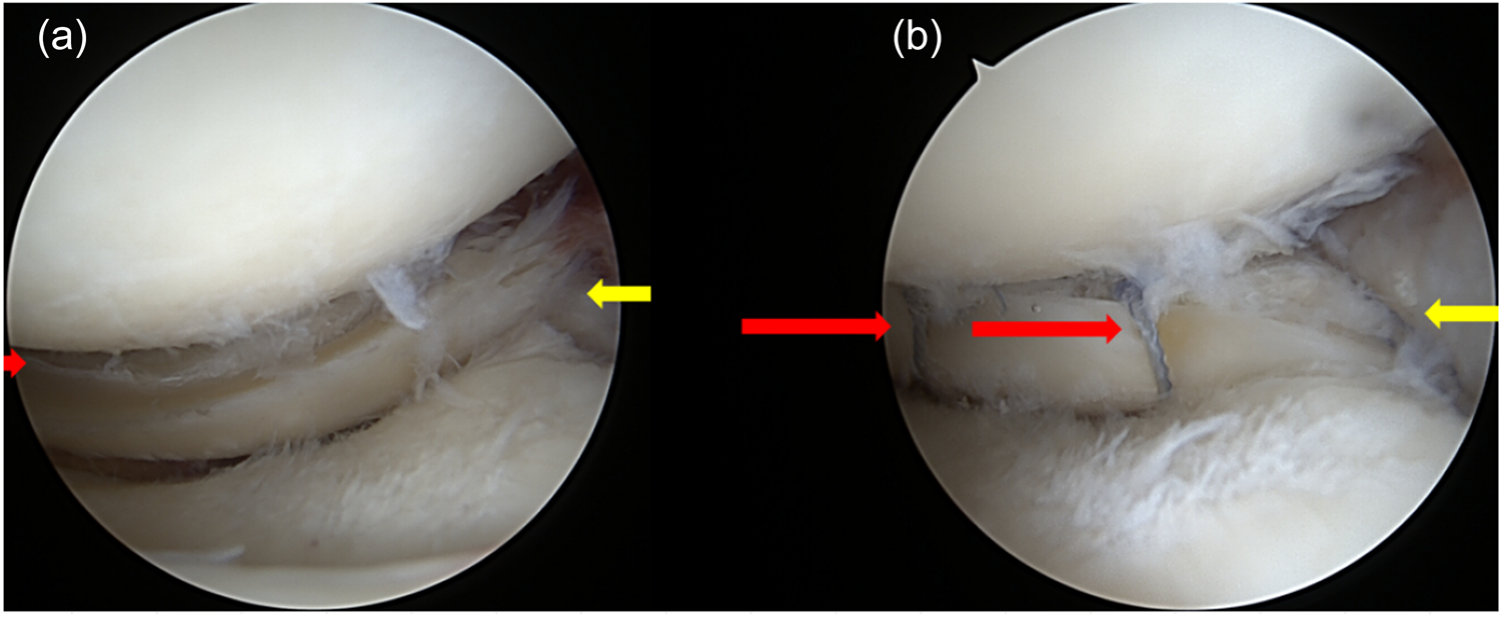
Arthroscopic evaluation of T‐shaped tears. (a) Arthroscopic assessment of tear pattern (red arrow – long T segment involving the posterior meniscal horn, yellow arrow – short T segment involving the meniscal root). (b) T‐shaped tear after all‐inside suture repair of both segments (long segment – red arrows, short segment – yellow arrow).

Longitudinal or “T” shaped tears (Group II) were repaired with all‐inside sutures with an additional transtibial pull‐out suture in cases of added root avulsion (Figure 4).

**Figure 5:**
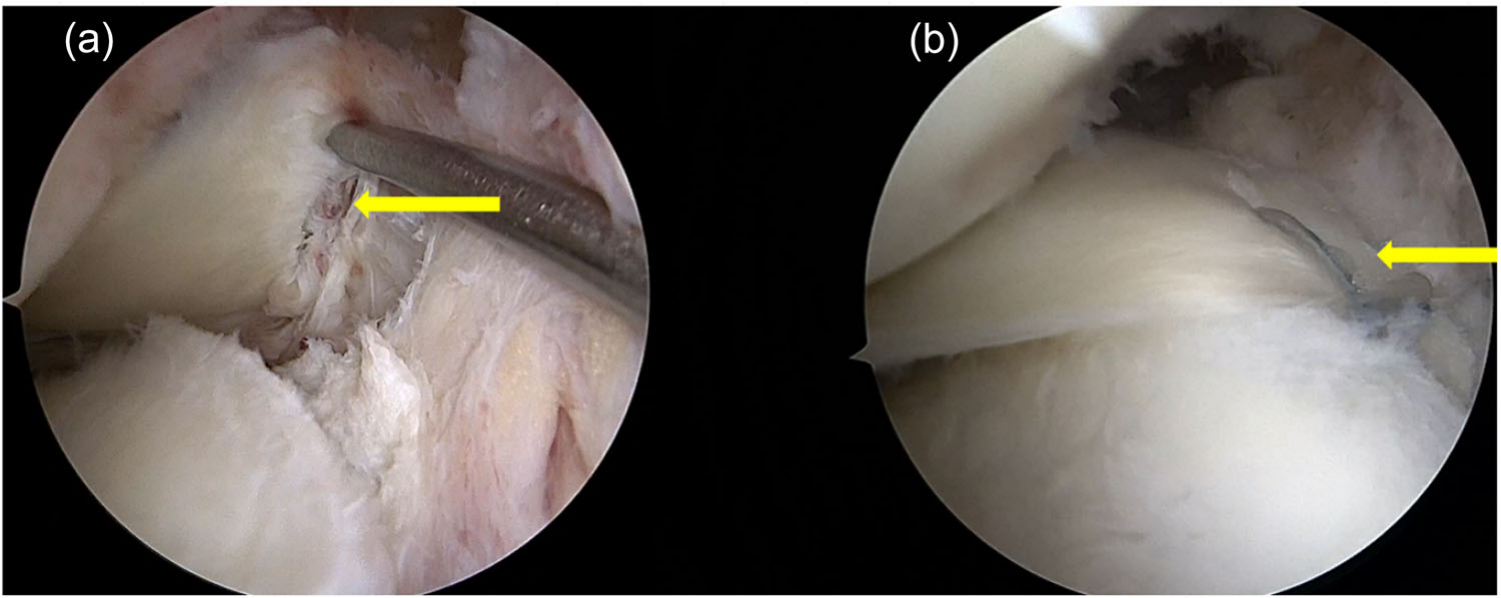
Arthroscopic evaluation of avulsion tears. (a) Arthroscopic assessment of an avulsion tear pattern (yellow arrow). (b) Avulsion tear after all‐inside repair (yellow arrow).

Soft tissue or bony avulsion of the root was repaired with the transtibial pull‐out repair technique (Figure 5). Specifically, an inverted vertical locking loop suture tape, was tied over a button to fix the root [20].

### Post‐operative rehabilitation

Post‐operatively, the operated knee was secured with a functional brace for 8 weeks. Range of motion was limited to 0°–60° for Weeks 1–3 and 0°–90° for Weeks 4–6. Touch‐down weight bearing was applied for 6 weeks. After 6 weeks the patient was allowed to gradually increase range of motion and weight bearing, as tolerated, to obtain normal gait and motion at 8 weeks. Quadriceps activation and strengthening exercises commenced on Day 1 after surgery and the patient followed a physiotherapy regime for 6–9 months. Jogging and squats were not allowed for 4 months.

### Statistical analysis

Statistical analysis was performed using SPSS software version 29.0 (IBM‐SPSS, New York, USA). Descriptive statistics and frequencies were applied to determine means, standard deviations, medians, range and frequencies. Propensity Score Matching (PSM) with a 1:2 ratio was performed, at a set calliper width of 0.5, using the following matching variables: age, gender, laterality, graft type, presence of LET, degree of medial meniscus involvement, and follow‐up. Balance diagnostics was conducted using the standardised mean difference (SMD) of 0.2–0.5 as threshold [44]. Normal distribution of the collected continuous variables was assessed using the Shapiro–Wilk test, together with descriptive statistics and graphical, qualitative, verification. The Wilcoxon signed‐rank test was used to compare pre‐operative and post‐operative continuous or ordinal outcomes, including KT‐1000 measurements, Lysholm score, IKDC score, and Tegner activity level. Pivot shift grades were analysed as ordinal data. Fisher's exact test was used to assess associations between treatment groups and categorical variables, including the presence of a LFNS and pMFL. Between‐group comparisons at each time point (preop. and postop.) were performed using the Mann–Whitney *U* test for two‐group comparisons or the Kruskal–Wallis test for more than two groups. Repeated‐measures differences in Tegner scores across follow‐up time points were assessed using the Friedman test. Differences in LME values between groups were evaluated using one‐way analysis of variance (ANOVA). Bonferroni correction for multiple comparisons was applied whenever possible. Statistical significance was set at *p* < 0.05. The level of significance was set at *p* < 0.05.

Because this was a retrospective cohort study, no a priori sample size calculation was performed. Nonetheless, due to the low sample size in each subgroup, effect size (Hedge's *g*) for both stability and PROMs results was computed.

## RESULTS

### Participants

After performing PSM the control and study groups had similar demographic, injury and treatment characteristics (Table 1).

**Table 1:** Demographic data.

Demographic data	Study group (ACLR and LMPRT repair), *n* = 41	Control group (isolated ACLR), *n* = 20	*p‐*value*
Age (year) mean ± SD (range)	23.78 ± 8.98 Range: 37	23.10 ± 9.14 Range: 36	0.67
Gender	1. Male: 58.5% 2. Female: 41.5%	Male: 60.0% Female: 40.0%	0.91
Laterality	1. Right: 53.7% 2. Left: 46.3%	Right: 55.0% Left: 45.0%	0.92
Graft	1. Quadriceps: 48.8% 2. Patella: 26.8% 3. Hamstrings: 24.4%	Quadriceps: 55.0% Patella: 20.0% Hamstrings: 25.0%	0.83
Distribution of LET	Yes: 68.3% No: 31.7%	Yes: 70.0% No: 30.0%	0.89
Medial meniscus	1. Normal: 53.7% 2. Repair: 36.6% 3. Meniscectomy: 9.8%	Normal: 60.0% Repair: 30.0% Meniscectomy: 10.0%	0.87
Follow up (months) mean ± SD (range)	44.73 ± 11.43 Range: 42	44.55 ± 12.37 Range: 42	0.98

Abbreviations: ACLRs, anterior cruciate ligament reconstructions; LET, Lateral extra‐articular tenodesis; LMPRT, lateral meniscus posterior root tear; SD, standard deviation.

* For categorical variables, the Fischer′s Exact test was used, while for continuous variables, the Mann–Whitney *U* test was performed.

### Knee instability assessment

Preoperatively, there were no significant differences in terms of pivot shift (*p* = 0.093) and KT‐1000 (*p* = 0.926) measurements between the study and control groups. No significant differences were found post‐operatively for pivot shift (*p* = 0.722) or KT‐1000 (*p* = 0.944). However, both groups demonstrated significant improvement (*p* < 0.001) post‐operatively in pivot shift grades and KT‐1000. Range, mean values and standard deviation of pivot shift and KT‐1000 measurements are presented in Table 2.

**Table 2:** Pre‐ and post‐operative Knee instability for study and control group.

	Pivot shift range (mean ± SD)		*p‐*value*	KT‐1000 range (mean ± SD)		*p‐*value
	Pre‐operative	Post‐operative		Pre‐operative	Post‐operative	
LMPRT group	1–3 (1.85 ± 0.79)	0–1 (0.07 ± 0.26)	*p* < 0.001	4–11 mm (7.00 ± 2.15)	0–2 (0.54 ± 0.74)	*p* < 0.001
Control group	1–3 (1.50 ± 0.68)	0–1 (0.10 ± 0.30)	*p* < 0.001	4–11 mm (6.90 ± 1.97)	0–2 (0.50 ± 0.68)	*p* < 0.001
*p‐*value	*p* = 0.093	*p* = 0.722		*p* = 0.926	*p* = 0.944	

Abbreviations: LMPRT, lateral meniscus posterior root tear; SD, standard deviation.

Significant post‐operative improvements in knee instability indexes were also noted in all subgroups. Table 3 summarises the range, mean values and standard deviation of pivot shift and KT‐1000 measurements for subgroups I, II and II.

**Table 3:** Knee instability pre‐ and post‐operatively for LMRT subgroups.

	Pivot shift range (mean ± SD)		*p‐*value*	KT‐1000** range (mean ± SD)		*p‐*value*
	Pre‐operative	Post‐operative		Pre‐operative	Post‐operative	
Subgroup I (*n* = 18)	1–3 (1.78 ± 0.80)	0–1 (0.6 ± 0.23)	*p* < 0.001	4–11 (7.22 ± 2.21)	0–2 (0.67 ± 0.84)	*p* < 0.001
Subgroup II (*n* = 9)	1–3 (1.78 ± 0.83)	0–1 (0.11 ± 0.33)	*p* = 0.007	4–11 (6.78 ± 2.22)	0–1 (0.33 ± 0.5)	*p* = 0.008
Subgroup III (*n* = 14)	1–3 (2 ± 0.78)	0–1 (0.7 ± 0.26)	*p* < 0.001	4–11 (6.86 ± 2.17)	0–2 (0.5 ± 0.76)	*p* < 0.001

*Note*: Subgroup I: Pivot shift results *g* = 2; KT‐1000 *g* = 3.9; Subgroup II: Pivot shift results *g* = 2.64; KT‐1000 *g* = 4; Subgroup III: Pivot shift results *g* = 2.23; KT‐1000 *g* = 3.91.

Abbreviations: LMPRT, lateral meniscus posterior root tear; SD, standard deviation.

Results of the Kruskal–Wallis H test indicated that there was no statistically significant difference across the four patient groups (three subgroups and control group) in pre‐operative pivot shift values (*H*(3) = 3.649, *p* = 0.302) and KT‐1000 values (*H*(3) = 0.391, *p* = 0.942). Likewise, no statistically significant difference in knee instability were also found post‐operatively between the four groups (pivot shift, *H*(3) = 0.369, *p* = 0.946 and KT‐1000, *H*(3) = 0.872, *p* = 0.832).

### Patient‐reported outcome measures (PROMs)

No significant differences were found preoperatively between the study and control group in Lysholm (*p* = 0.238), IKDC (*p* = 0.192) and Tegner (*p* = 0.762) scores. Similarly, post‐operative scores had comparable results (Lysholm *p* = 0.919, IKDC *p* = 0.871, Tegner *p* = 0.912). Nevertheless, significant (*p* < 0.001) improvements in all clinical scores were noticed for both the study and control group. Range, mean values and standard deviation of Lysholm, IKDC, and Tegner scores are presented in Table 4.

**Table 4:** Patient reported outcome measures for study and control group.

	Lysholm range (mean ± SD)		*p‐*value	IKDC range (mean ± SD)		*p‐*value	Tegner range (mean ± SD)		
	Pre‐operative	Post‐operative		Pre‐operative	Post‐operative		Pre‐operative	Post‐operative	*p‐*value
LMPRT group	42–72 (62.63 ± 8.09)	83–100 (95 ± 4.84)	*p* < 0.001	40.2–70.1 (60.72 ± 8.06)	79.3–100 (91.98 ± 5.50)	*p* < 0.001	1–3 (2.10 ± 0.76)	4–9 (6.17 ± 1.70)	*p* < 0.001
Control group	36–71 (58.40 ± 11.18)	83–100 (94.80 ± 5.55)	*p* < 0.001	35.7–68.9 (56.37 ± 11.49)	78.1– 100 (92.04 ± 6.31)	*p* < 0.001	1–3 (2.15 ± 0.87)	4–9 (6.10 ± 1.74)	*p* < 0.001
*p‐*value	*p* = 0.238	*p* = 0.919		*p* = 0.192	*p* = 0.871		*p* = 0.762	*p* = 0.912	

Abbreviations: IKDC, International Knee Documentation Committee; LMPRT, lateral meniscus posterior root tear; SD, standard deviation.

All subgroups showed significant improvement post‐operatively in Lysholm, IKDC, and Tegner scores. Table 5 presents the range, mean values, and standard deviation of Lysholm, IKDC and Tegner scores in subgroups I, II and III.

**Table 5:** Patient reported outcome measures for subgroups.

	Lysholm range (mean ± SD)		*p‐*value	IKDC range (mean ± SD)		*p‐*value
	Pre‐operative	Post‐operative		Pre‐operative	Post‐operative	
Subgroup I (*n* = 18)	54–72 (64.17 ± 6.60)	88–100 (94.89 ± 4.01)	*p* < 0.001	51.8–70.1 (62.12 ± 6.63)	85.0–100 (92.69 ± 4.52)	*p* < 0.001
Subgroup II (*n* = 9)	42–72 (61.56 ± 9.22)	83–100 (95.56 ± 6.28)	*p* = 0.008	40.2–70.1 (59.77 ± 9.14)	79.3–100 (92.32 ± 7.82)	*p* = 0.008
Subgroup III (*n* = 14)	42–72 (61.36 ± 9.30)	88–100 (94.79 ± 5.26)	*p* < 0.001	40.2‐68.9 (59.52 ± 9.29)	85–100 (90.85 ± 5.17)	*p* < 0.001

*Note*: Subgroup I: Lysholm results *g* = 5.6; IKDC *g* = 5.38; Subgroup II: Lysholm results *g* = 4.3; IKDC *g* = 3.82; Subgroup III: Lysholm results *g* = 4.42; IKDC *g* = 4.16.

Abbreviations: IKDC, International Knee Documentation Committee; SD, standard deviation.

The comparison between the three subgroups and of the control group to each of the subgroups revealed no significant differences preoperatively in Lysholm (*H*(3) = 2.348, *p* = 0.503), IKDC (*H*(3) = 2.459, *p* = 0.483), and Tegner (*H*(3) = 1.038, *p* = 0.792) scores. Similarly, these groups of patients had comparable Lysholm (*H*(3) = 0.827, *p* = 0.843), IKDC (*H*(3) = 0.965, *p* = 0.810), and Tegner (*H*(3) = 1.057, *p* = 0.788) scores post‐operatively.

### Lateral meniscus extrusion (LME)

Pre‐operative LME ranged from 1.22–4.74 mm (2.50 ± 0.89) in the study group and 0.04–1.79 mm (0.80 ± 0.51) in the control group, *p* < 0.001.

Analysing differences in pre‐operative extrusion among the four groups revealed a significant overall effect, *F*(3,57) = 25.21, *p* < 0.001. All three LMPRT groups had significantly greater extrusion than the control group (*p* < 0.05), while no significant differences were found between the tear types themselves. Mean extrusion was lowest in the control group (0.81 ± 0.51 mm) compared with the Oblique Radial tear (2.57 ± 1.03 mm), T‐type tear (1.92 ± 0.74 mm) and avulsion injury groups (2.79 ± 0.61 mm).

A second one‐way ANOVA examined differences in extrusion reduction at follow‐up among the three LMPRT groups. No significant differences were identified, *F*(2, 38) = 2.65, *p* > 0.05. Mean extrusion reduction was 1.24 ± 0.56 mm in the Oblique Radial tear group, 0.74 ± 0.43 mm in the T‐type tear group and 1.08 ± 0.56 mm in the Avulsion injury group.

### LMPRT healing

No failures of lateral meniscus posterior root tear healing were found in the study group at a minimum follow‐up of one year. The results of complete and partial healing of the study group and the subgroups are presented in Table 6.

**Table 6:** Healing rate of lateral meniscus posterior root tear.

	Complete healing	Partial healing
Subgroup I	12 (66.7%)	6 (33.3%)
Subgroup II	7 (77.8%)	2 (22.2%)
Subgroup III	12 (85.7%)	2 (14.3%)
Study group (total)	31 (75.6%)	10 (24.4%)

Results of the Kruskal–Wallis H Test indicated that there was no statistically significant difference in healing levels across the three meniscal tear groups, *H*(2) = 1.54, *p* = 0.463.

### Presence of a LFNS and pMFL

Fisher's Exact test indicated that there were no significant associations between the study and control group neither for the presence of the LFNS (*p* = 0.249), nor for the presence of pMFL (*p* = 0.353). The Chi‐square statistic found no correlations between the three subgroups and the presence of LFNS (*p* = 0.62); likewise, no correlation was found for the presence of pMFL (*p* = 0.47). The presence of LFNS and pMFL in the study and control groups is presented in Table 7.

**Table 7:** Presence of LFNS and pMFL on pre‐operative MRI.

	LFNS	pMFL
Subgroup I	2 (11.1%)	6 (33.3%)
Subgroup II	1 (11.1%)	2 (22.2%)
Subgroup III	3 (21.4%)	2 (14.3%)
Entire study group	6 (14.6%)	10 (24.4%)
Control group	1 (5%)	17 (85%)

Abbreviations: LFNS, lateral femoral notch sign; MRI, magnetic resonance imaging; pMFL, posterior meniscofemoral ligament.

## DISCUSSION

The most important finding of the present study is that a LMPRT repair performed concurrently with ACLR results in improved stability testing and PROMs, similar to ACLR alone. Furthermore, dividing the LMPRT into their different subgroups did not seem to have an impact on stability testing or PROMs improvement following surgery. These findings add to the growing evidence that restoring LMPR integrity is important for optimal post‐operative function in knees that have undergone ACL reconstruction.

Preoperatively, patients with LMPRT exhibited greater anterior–posterior and rotational instability than those with isolated ACL deficiency. This finding is consistent with previous biomechanical and clinical studies demonstrating that LMPRT increases ALRI and amplifies the magnitude of the pivot shift [28, 36]. Despite these deficits, both pivot‐shift grades and KT‐1000 values normalised following combined ACL and LMPRT repair, with outcomes comparable to isolated ACLR. Similar improvements in knee stability have been reported, with LMPR repair restoring knee kinematics and showing no significant side‐to‐side differences in KT‐1000 measurements at a minimum follow‐up of 2 years [42]. Nonetheless, one should be aware of the inconsistent role that LMPRT play over rotatory laxity on quantitative pivot‐shift analyses, as it has been suggested that LMPRT might not always result in greater rotatory laxity [26]. However, the present findings align more closely with earlier clinical and cadaveric research demonstrating that root deficiency compromises rotation stability and that surgical repair effectively restores it. The generally favourable stability outcomes in the present study may also be attributed to the use of LEAP in a subset of patients.

Despite each type of LMPRT imposing different surgical challenges, all LMPRT demonstrated significant post‐operative improvements in PROMs, consistent with previous studies showing that LMPR repair combined with ACLR yields satisfactory Lysholm, IKDC, and Tegner scores [2, 4, 21, 24, 31, 32, 37, 42, 46]. Most importantly, LMPR repair does not appear to impair functional recovery compared with isolated ACLR. Therefore, the present study supports routine repair of LMPRT, as it results in equivalent or near‐equivalent functional outcomes when patients undergoing LMPR repair are compared with those undergoing ACLR with an intact lateral meniscus, consistent with findings reported in the recent literature [9, 40, 41].

With the presence of a LMPRT, LME becomes a well‐established indicator of root integrity. The present study confirm that pre‐operative LME is significantly increased in LMPRT compared with isolated ACL injuries, consistent with prior reports showing large extrusion values in complete LMPRT and minimal extrusion in intact or partially torn roots [16]. Usually, a pre‐operative LME ≥ 2.2 mm has been identified as a strong predictor of complete LMPRT in ACL‐injured knees [43]. These results align precisely with the LME values observed in the presently‐reported cohort. However, post‐operative LME values at follow‐up present a less clear picture. Although they did not differ between LMPRT groups, they remained slightly higher than those observed in the control group. This result might also be due to the relatively small sample size. Prior literature supports the expectation of partial but meaningful reduction in LME values following LMPR repair [31], while at the same time signalling that hoop stress normalisation might not always be obtained [1, 12].

Despite difficulties in optimising post‐operative hoop stresses following LMPRT repair, healing rates do seem favourable across reported literature [45]. Although second‐look arthroscopy was not available in the present study, MRI‐based healing rates proved to be favourable at final follow‐up. Importantly, complete and partial healing should be differentiated [42]. Another factor influencing LMPR healing is the pMFL and its ability to alleviate load on the LMPR during ROM. Thus, in the absence of the pMFL, healing may be impaired, particularly in the context of early active ROM rehabilitation. Prior biomechanical studies indicate that the meniscofemoral ligaments can stabilise the lateral meniscus and protect against extrusion, although their presence and morphology can vary [10, 18, 33]. This further suggests that pre‐operative evaluation of the pMFL integrity could help stratify and evaluate post‐operative LMPRT repair healing potential.

The present findings confirm the high prevalence of a deep LFNS in LMPRT, consistent with earlier work demonstrating that LFNS is strongly associated with combined ACL and LMPR injuries. Thus, the deep LFNS remains and important radiographic marker of a likely high energy rotatory injury component [5], signalling a possible posterolateral compartment involvement and increased lateral posterior tibial slope [7].

The present study has several limitations. It is retrospective in nature, which may introduce selection and measurement bias. The sample size is relatively small, particularly within each LMPRT subgroup, limiting the statistical power to detect subtle intergroup differences. For this reason, caution is recommended when interpreting these findings. Meniscal healing was assessed using MRI rather than second‐look arthroscopy, which, while clinically practical, is less accurate for evaluating tissue integrity. Additionally, concomitant procedures such as a lateral tenodesis may have influenced post‐operative stability outcomes. At the time when these specific procedures were performed, there was no consensus on clear indications for LEAP [38]. Lastly, the 2‐year follow‐up duration might be considered too short to detect function disturbances or arthritis progression compared to ACLR alone.

## CONCLUSION

In ACL‐injured patients, LMPRT significantly worsen pre‐operative knee stability and increase meniscal extrusion. However, LMPRT repair performed at the time of ACLR effectively restores stability, reduces extrusion, and results in functional outcomes comparable to isolated ACLR. Tear morphology does not appear to influence post‐operative clinical or MRI outcomes. These findings reinforce the importance of early recognition and repair of LMPRT and support the continued refinement of diagnostic imaging markers and repair techniques.

## AUTHOR CONTRIBUTIONS

**Nikolaos Koukoulias:** Conceptualisation; methodology; writing—original draft writing—review and editing; supervision. **George Mihai Avram:** Writing—original draft writing—review and editing; validation. **Evangelia Germanou:** Data curation; formal analysis; software. **Dimitris Koukoulias:** Investigation; data curation; resources. **Georgios Graikos:** Investigation. **Angelo Vasiliadis:** Investigation. Ioannis Tsifountoudis: Data curation. **Theofilos Dimitriadis:** Writing—review; project administration.

## CONFLICT OF INTEREST STATEMENT

The authors declare no conflicts of interest.

## DECLARATION

AI was used for syntax and grammar optimisation.

## ETHICS STATEMENT

General Consent was signed by all included patients. Ethical approval and institutional review board (IRB) approval was obtained at Saint Luke′s Hospital, registration number E.E./10‐03‐2025.

## Data Availability

Data is available on reasonable request by contacting the corresponding author.
